# Time to blood culture positivity as a predictor of clinical outcome in patients with *Candida albicans* bloodstream infection

**DOI:** 10.1186/1471-2334-13-486

**Published:** 2013-10-20

**Authors:** Cintia Zoya Nunes, Alexandre R Marra, Michael B Edmond, Elivane da Silva Victor, Carlos Alberto Pires Pereira

**Affiliations:** 1 Infectious Diseases Division, Universidade Federal de São Paulo (UNIFESP), São Paulo, Brazil; 2 Division of Medical Practice, Hospital Israelita Albert Einstein, São Paulo, Brazil; 3 Department of Internal Medicine, Virginia Commonwealth, University School of Medicine, Richmond, Virginia, USA

**Keywords:** Candida, Bloodstream infection, Time to positivity, Antifungal therapy

## Abstract

**Background:**

Few studies have assessed the time to blood culture positivity as a predictor of clinical outcome in fungal bloodstream infections (BSIs). The purpose of this study was to evaluate the time to positivity (TTP) of blood cultures in patients with *Candida albicans* BSIs and to assess its impact on clinical outcome.

**Methods:**

A historical cohort study with 89 adults patients with *C. albicans* BSIs. TTP was defined as the time between the start of incubation and the time that the automated alert signal indicating growth in the culture bottle sounded.

**Results:**

Patients with BSIs and TTPs of culture of ≤36 h (n=39) and >36 h (n=50) were compared. Septic shock occurred in 46.2% of patients with TTPs of ≤36 h and in 40.0% of patients with TTP of >36 h (p=0.56). A central venous catheter source was more common with a BSI TTP of ≤36 h (p=0.04). Univariate analyis revealed that APACHE II score≥20 at BSI onset, the development of at least one organ system failure (respiratory, cardiovascular, renal, hematologic, or hepatic), SOFA at BSI onset, SAPS II at BSI onset, and time to positivity were associated with death. By using logistic regression analysis, the only independent predictor of death was time to positivity (1.04; 95% CI, 1.0-1.1, p=0.035), with the chance of the patient with *C. albicans* BSI dying increasing 4.0% every hour prior to culture positivity.

**Conclusion:**

A longer time to positivity was associated with a higher mortality for *Candida albicans* BSIs; therefore, initiating empiric treatment with antifungals may improve outcomes.

## Background

Candidemia is a serious problem in tertiary hospitals all over the world [1-3]. Despite a relatively low incidence, it is associated with higher rates of morbidity and mortality [1-3].

The use of automated systems for blood cultures was one of the great advances in the diagnosis of candidemia [4,5]. This method offers improved sensitivity, which is estimated to be approximately 70% [5]. Currently, it appears that the number of these infections has stabilized, although their mortality remains unchanged [1-3,5].

*Candida albicans* is still the primary cause of these infections, representing on average half of the cases [1,6]. The increase in non-albicans species, carries therapeutic implications because the pattern of susceptibility to azoles is species-specific [3,6].

As with all infectious processes, rapid diagnosis of fungal infection combined with appropriate treatment reduces early mortality [5-7]. Blood cultures, still considered the gold standard in the diagnosis of these infections, can take days to turn positive and even longer to identify the species [5,6,8].

Time to blood culture positivity (TTP) has recently been used diagnostically and prognostically for bacteremia [9-11]. Given the paucity of data on TTP in BSI by *Candida* species, the present study aims at better understanding it and its impact on clinical outcome.

## Methods

### Setting

Hospital São Paulo, at the Universidade Federal de São Paulo is a 700-bed tertiary-care facility in São Paulo, Brazil. The hospital houses four intensive care units (ICUs), including a pediatric ICU. This study was approved by the Ethics Committee of the Universidade Federal de São Paulo.

### Study design

Patients with BSIs from 1 January 2002 through 31 July 2009 were identified retrospectively by use of the electronic medical microbiology record. For each case, the time to blood culture positivity was retrieved from the hospital's automated blood culture instrument. The medical microbiology record identified the patient by medical record number so that a retrospective chart review could be conducted. Patients were considered to have had a BSI due to *Candida* spp if one or more blood cultures were positive for this organism. Each patient was included only once, at the time of the first BSI. Patients less than 18 years old, those with polymicrobial infections, and those receiving antifungal therapy at the time of the BSI were excluded from the analysis.

Data collected included age; gender; location of the patient (ward *versus* ICU); the duration of hospitalization prior to the onset of the BSI; the presence of predisposing clinical factors, including neutropenia (defined as an absolute neutrophil count of <500/μl); the use of peritoneal dialysis or hemodialysis; and the presence of central venous catheters. The sources of secondary BSIs were identified by cultures of samples obtained from the primary site of infection that yielded the same pathogen. Adverse outcomes (organ failure and in-hospital mortality) occurring during the course of hospitalization were recorded.

The severity of the underlying disease preceding the positive blood culture was classified by use of the Charlson weighted comorbidity índex [12]. The patient's physiological condition on the day of the BSI was assessed by using the APACHE II score [13], SAPS II [14] and the SOFA score [15]. At the onset of the BSI, the clinical condition of each patient was classified as systemic inflammatory response syndrome (SIRS), sepsis, severe sepsis, or septic shock using criteria previously published by the American College of Chest Physicians/Society of Critical Care Medicine [16]. SIRS was defined as two or more of the following: (i) a temperature of >38°C or <36°C, (ii) a heart rate of >90 beats per minute, (iii) a respiratory rate of >20 breaths per minute or a partial arterial CO_2_ pressure of <32 mm Hg, or (iv) a white blood cell count of >12 ×10^9^/liter or <4 ×10^9^/liter or the presence of more than 10% immature neutrophils.

Severe sepsis was defined as organ dysfunction, hypotension, or systemic manifestations of hypoperfusion. Septic shock was defined as sepsis associated with hypotension unresponsive to intravenous fluid challenge or the need for treatment with a vasopressor agent. The presence of organ system failure at the time of the BSI and during the clinical course was assessed by using the criteria described by Fagon et al. [17]. Nosocomial infection was defined as an infection that occurred >48 h after hospital admission, an infection that occurred <48 h after admission to the hospital for patients who had been hospitalized in the 3 weeks prior to the admission, or an infection that occurred <48 h after admission to the hospital for patients who had been transferred from another hospital or nursing home [18]. The sources of infection were also defined according to Centers for Disease Control and Prevention criteria [18]. Time to positivity was defined as the time between the start of incubation and the time to sounding of the alert signal on the automated blood culture instrument. Adequate empirical antifungal treatment was defined as therapy that was administered within 24 h after samples for blood culture were obtained that included any antifungal agent to which *Candida albicans* was susceptible [6].

### Microbiological methods

Blood cultures were processed by the institution's clinical laboratory using the BACTEC®9240 blood culture instrument (Becton Dickinson, Maryland, EUA). Each blood culture set consisted of an FA aerobic bottle and an SN anaerobic bottle. All the samples of blood cultures were collected and submitted in a timely manner to the microbiology laboratory. All cultures were obtained via peripheral venipuncture and at least two bottles were obtained for each patient. The bottles were loaded into the instrument (24 h a day, 7 days a week) without delay at any time of the day. The time to positivity of the first bottle in a set to be flagged as positive was used to determine the time to positivity and was obtained by using the system's software.

The confirmation of the species was performed by screening for *C. albicans* using CHROMagar Candida ®-(CHROMagar Microbiology, Paris, France). Samples of non-albicans *Candida* were identified by biochemical profile, the manual method ID 32 C® (BioMérieux, Marcy-l'Étoile, France) and supplemented by analysis of subculture microculture. Initial identification of *C. albicans* by CHROMagar test - Candida® was confirmed by the presence of chlamydoconidia in microculture.

### Statistical analysis

Continuous variables were compared by using the Student *t* test for normally distributed variables and the Mann–Whitney U test for nonnormally distributed variables. Differences in proportions were compared by a chi-square test or Fisher's exact test, when appropriate. Mean values ±1 standard deviation were reported. Alpha was set equal to 0.05, and all tests of significance were two tailed. When collinearity was identified between two variables in a correlation matrix, the one with the greatest clinical relevance associated with mortality was included in the multivariate analysis. Odds ratios (ORs) with 95% confidence intervals (CIs) were calculated for all variables. Variables found to be significant by univariate analysis were then entered into a multivariate model. All statistical analyses were done by using the Statistical Package for the Social Sciences software (SPSS, Inc., Chicago, IL).

## Results

### Study population and patient characteristics

A total of 272 patients with episodes of *Candida* spp BSIs were identified at Hospital São Paulo during the 7-year study period. Of these, 101 patients were excluded; 14 patients were on antifungal therapy when blood samples for culture were obtained, 22 patients because they had polymicrobial BSIs, and 65 due to incomplete data in medical records. Of the remaining 171 patients, 89 had *Candida albicans* BSI and were further analyzed. The other 82 patients had infection due to non-albicans species.

The mean age of patients with *Candida albicans* BSI was 62.5 ± 16.5 years (range, 18 to 93 years). Fifty-seven patients (57.3%) were over 60 years of age. The most frequent diagnoses responsible for hospitalization were gastrointestinal diseases (27.0%), respiratory diseases (21.4%), and solid and hematologic malignancies (12.3%). The most frequent sources of BSIs were central venous catheters (42.7%) and gastrointestinal (33.7%). Most BSIs (70.8%) occurred after 21 days of hospitalization (Table 1).

**Table 1:** **Demographic characteristics of 89 patients with *C. albicans *BSI**

**Variables**	**N**	**%**
**Gender**		
Male	54	60.7
Female	35	39.3
**Age (years)**		
<60	38	42.7
≥60	51	57.3
**ICU**	20	22.5
**Diagnosis on admission**		
Gastrointestinal disease	24	27.0
Respiratory disease	19	21.4
Neoplasm	11	12.3
Neurologic disease	6	6.8
Cardiovascular disease	5	5.6
Trauma	4	4.5
Renal failure	4	4.5
AIDS	2	2.2
Other	14	15.7
**Site of infection**		
Catheter	38	42.7
Abdominal	30	33.7
Respiratory	9	10.1
Urinary	8	9.0
Other	4	4.5
**Hospital stay prior to BSI (days)**		
≤2*	1	1.1
3-7	4	4.5
8-14	11	12.3
15-21	10	11.3
>21	63	70.8
**Time to positivity**		
≤24 hs	16	18.0
>24 --≤36 hs	23	25.8
>36--≤48 hs	20	22.5
>48hs	30	33.7
**Receiving adequate antifungal therapy**		
No	69	77.5
Yes	20	22.5

### Time to positivity

The mean time to positivity was 41.9 h ± 19.2 hours (range 6.7 to 95.2 hours) for *Candida albicans*. In approximately half of the cases (56.2%) growth of *Candida albicans* occurred within 36 hours. We therefore divided the cases into two groups: patients with an early time to positivity (TTP of ≤36 h) and patients with a late time to positivity (TTP of >36 h). Associated risk factors and outcomes of the two TTP groups are summarized in Table 2.

**Table 2:** **Univariate analysis of factors associated with time to positivity in *C. albicans *BSIs**

**Variables**	** Time to positivity ≤36 hs (n=39)**		** Time to positivity >36 hs (n=50)**		**P**
	**N**	**%**	**N**	**%**	
**Demographic characteristics**					
**Age, mean years ±SD**	58.4±17.8		65.6±14.8		0.04
**Male**	25	64.1	29	58.0	0.56
**Underlying conditions**					
**Charlson ≥3**	23	59.0	26	52.0	0.51
**Rapidly fatal disease (MacCabe)**	26	66.7	28	56.0	0.31
**Diabetes**	6	15.4	13	26.0	0.23
**Neoplasia**	16	41.0	10	20.0	0.03
**Neutropenia**	1	2.6	2	4.0	0.44
**Surgery**	24	61.5	26	52.0	0.34
**Blood transfusion**	21	53.8	15	30.0	0.02
**Conditions related to the clinical course**					
**APACHE II score ≥20 at BSI onset**	11	28.2	23	46.0	0.09
**Adequate antifungal therapy**	13	33.3	7	14.0	0.03
**Source of infection**					
**Intravascular catheter**	22	56.4	16	32.0	0.04
**Gastrointestinal**	7	17.9	23	46.0	0.01
**Respiratory tract**	5	12.8	4	8.0	0.45
**Urinary tract**	3	7.7	5	10.0	0.71
**Other**	2	5.2	2	4.0	0.83
**Outcomes**					
**Organ failure**	31	79.5	38	76.0	0.70
**Septic shock**	18	46.2	20	40.0	0.56
**Respiratory failure**	26	66.7	36	72.0	0.59
**Renal failure**	15	38.5	18	36.0	0.81
**Hematologic failure**	1	2.6	4	8.0	0.26
**Hepatic failure**	3	7.7	5	10.0	0.71
**In-hospital mortality**	29	74.4	41	82.0	0.38

Neoplasia was more commonly associated with a BSI TTP of ≤36 h than with a TTP of >36 (41.0% and 20.0%, respectively; *P* = 0.030). Blood transfusion was also more commonly associated with a BSI TTP of ≤36 h (53.8.0% vs. 30.0%; *P* = 0.023). Central venous catheters were more commonly associated with a BSI TTP of ≤36 h (56.4% vs. 32.0%; *P* < 0.04). There was also a statistically significant difference in the proportion of patients with gastrointestinal sources of BSIs between the two groups (17.9% in the early group vs. 46.0% in the late group, *P*=0.01*,* respectively). Patients with early positive cultures were more likely to have received adequate antifungal therapy (33.3% vs. 14.0%; *P* = 0.03).

### Clinical course

No statistically significant differences were observed in organ dysfunction and in-hospital mortality between the two groups (Table 2). There was a trend for patients with late positivity to have an APACHE II score≥20 at BSI onset (46.0% vs. 28.2%; *P*=0.09) (Table 2).

Univarate analysis revealed that APACHE II score≥20 at BSI onset, the development of at least one organ system failure (respiratory, cardiovascular, renal, hematologic, or hepatic), SOFA at BSI onset, SAPS II at BSI onset, and time to positivity were associated with death (Table 3). Age, gender, inadequate antifungal therapy, Charlson score of ≥3, and neoplasia were not significant predictors of mortality on univariate analysis.

**Table 3:** **Risk factors for hospital mortality in patients with *C. albicans *BSI**

**Variables**	** Died (n=70)**		** Recovered (n=19)**		** Univariate analysis**		** Multivariate analysis**	
	**N**	**%**	**N**	**%**	**OR**	**CI 95%**	**OR**	**CI 95%**
**Age >60 years**	43	61.4	8	42.0	2.2	0.78-6.13		
**Male gender**	45	64.3	9	47.4	0.5	0.18-1.39		
**APACHE II score** ≥**20 at BSI onset**	32	45.7	2	10.5	7.2	1.54-33.35	2.1	0.3-12.8
**Organ failure (at least one)**	59	84.2	10	52.5	4.8	1.60-14.60	2.9	0.6-13.2
**Respiratory failure**	54	77.1	8	42.1	4.6	1.60-13.50		
**Cardiovascular failure**	35	50.0	3	15.8	5.3	1.43-19.95		
**Renal failure**	29	41.4	4	21.0	2.7	0.80-8.82		
**Hematologic failure**	5	7.1	-	-	1.3	1.15-1.45		
**Hepatic failure**	8	11.4	-	-	1.3	1.16-1.47		
**Inadequate antifungal therapy**	55	78.6	14	73.7	1.31	0.41-4.22		
**Charlson score ≥3**	40	57.1	9	47.3	1.5	0.54-4.10		
**Neoplasia**	20	28.5	6	31.5	0.9	0.29-2.60		
**SOFA at BSI onset, mean (±SD)**	6.7 (4.3)		3.6 (2.3)		1.28	1.07-1.52	1.05	0.8-1.3
**SAPS II at BSI onset, mean (±SD)**	48.4 (15.4)		35.2 (14.3)		1.06	1.02-1.11	1.04	0.9-1.1
**Time to positivity (in hours), mean (±SD)**	43.9 (19.8)		34.6 (15.4)		1.03	1.0-1.06	1.04	1.0-1.1

Using logistic regression analysis, the only independent predictor of death was time to positivity (1.04; 95% CI, 1.0-1.1), with mortality increasing 4.0% per hour prior to culture positivity (Table 3). A second model including inadequate antifungal therapy still showed that time to positivity was the only independent predictor of death (1.03; 95% CI 1.0-1.07)*.*

## Discussion

Recently it has been shown that shorter time to blood cultures positivity in automated systems has been associated with worse prognosis in infections caused by bacteria (i.e., shorter times are associated with higher mortality) [9-11]. This has been shown for *S. aureus*[9], *Streptococcus pneumoniae*[11] and *Escherichia coli* BSI [10]. These findings prompted us to study the time to positivity of candidemia (*C. albicans*), since the studies analyzing time of positive *Candida* spp culture are rare and almost all were performed in vitro [19-21].

In our study, only the time to blood culture positivity was predictive of mortality for *Candida albicans*. The severity of illness (APACHE II, SAPS II and SOFA) was not significantly different between the two groups.

We identified in the medical literature only three clinical studies that analyzed the time to positivity for candidemia, but these had different goals: comparing the differential time to positivity between specimens obtained peripherally and via a central venous catheter [22]; quantifying the time between the collection of blood culture and its positivity [8]; and evaluating the time to positivity for detecting *Candida* species resistant to fluconazole [23].

Regarding *Candida albicans* BSI, our study showed the longer the time to positivity, the higher the mortality. We found only one report with a similar finding and this was for *Staphylococcus aureus* bacteremia [24]. In this retrospective study they observed that a shorter time to positivity was associated with methicillin susceptibility and an endovascular source; multivariate analysis showed higher mortality both with time to positivity ≤ 12 or >48 hours [24].

Physicians often wait for the blood cultures to grow *Candida* prior to beginning antifungal therapy. However, even if the patient has a stable clinical condition and not receiving empirical antifungal treatment, this may be associated with a poor outcome.

Until the early 2000s, the vast majority of candidemia cases were diagnosed in intensive care units [3,25]. Recent studies have shown a decrease in the proportion of *Candida* BSIs occurring in ICUs, especially in Latin America countries, with a fall between the years 2008 and 2009 from 64.9% to 42.6% [26].

We observed that only 22.5% of patients with candidemia (*C. albicans*) received adequate antifungal therapy in the first 24 hours after the suspected infection or collection of blood cultures. Although the reason for not instituting empiric treatment was not evaluated, we believe it has occurred at least in part, due to the delay in blood culture positivity. Other studies have also found that candidemia has one of the highest rates of inadequate empirical treatment in the first 24 hours after suspected infection [6,7].

A multicenter study reported that the mean time for initiating empiric antifungal therapy was 3.8 days [3]. These data emphasize the need to consider early initiation of empiric therapy given that blood cultures are slow to turn positive for *Candida* species.

Comparing the mean time to positivity for *Candida albicans* with other studies, we found that the automated BacT/ALERT® time growth is faster [8,27]. Lai and colleagues (2012) reported similar time using the BACTEC® system [23]. It should be noted that in our study we did not use specific culture medium for fungi. However another study also used specific culture medium for fungi (BACTECTM Myco/F Lytic) whose time to positivity is known to be shorter, especially in the identification of non-albicans *Candida* species. They showed differences between the mean time to positivity for *Candida albicans* between the specific culture medium for fungi versus a non-specific medium for fungi (aerobic culture), 34 ± 25 h vs. 42 ± 19 h, respectively [23].

Our study is limited by the retrospective nature of our analysis. In addition, because of the relatively small sample size of our study (n=89), a type II error could have occurred, which would limit the ability to detect a statistically significant difference in the other variables considered as predictors of mortality.

## Conclusions

The present study showed that the time to positivity for *Candida albicans* BSI is associated with a significantly greater risk for mortality. These data suggest that physicians should consider empiric antifungal therapy in patients with risk factors for candidemia rather than waiting for the growth of *Candida albicans* in culture.

## Competing interests

The authors have declared that no competing interests exist.

## Authors’ contributions

CZN, ARM, and ESV participated in the data collected and data analysis. ARM, MBE and CAPP participated in the design and coordination. CZN, ARM, MBE, ESV and CAPP helped to draft the manuscript and to provide critical review of the manuscript. All authors read and approved the final manuscript.

## Pre-publication history

The pre-publication history for this paper can be accessed here:

http://www.biomedcentral.com/1471-2334/13/486/prepub
